# Risk factors for periodontal ligament area reduction in maxillary central incisors following orthodontic treatment: a retrospective analysis using a deep learning-based CBCT-PDL segmentation network

**DOI:** 10.1186/s12903-026-09294-3

**Published:** 2026-07-15

**Authors:** Ruiqi Li, Sha Su, Liping Zhan, Xiaochuan Fan, Xing Chen, Cai He, Xiaofeng Huang

**Affiliations:** Department of Stomatology, Beijing Friendship Hospital, Capital Medical University, No.95 Yong’an Road, Xicheng District, Beijing, 100050 China

**Keywords:** Periodontal ligament, Cone-beam computed tomography, Orthodontic treatment, Root length, Risk factors, Deep learning

## Abstract

**Objective:**

To analyze the relevant risk factors causing the reduction of periodontal ligament area (PDLA) of the maxillary central incisor by measuring the PDLA before and after orthodontic treatment using a previously validated cone-beam computed tomography-periodontal ligament (CBCT-PDL) deep learning segmentation network, providing a theoretical basis for orthodontic clinical treatment design.

**Methods:**

A retrospective analysis was performed on 63 patients who completed comprehensive orthodontic treatment in the Department of Stomatology, Beijing Friendship Hospital, Capital Medical University, between May 2021 and December 2022. Pre- and post-treatment CBCT images and clinical data were extracted from the hospital’s dedicated imaging database. Using the CBCT-PDL segmentation network and orthodontic measurement and analysis software, PDLA and root length (RL) of 126 maxillary central incisors, were quantified before and after orthodontic treatment. Absolute and percentage changes in PDLA (ΔPDLA and ΔPDLA%, respectively) and RL (ΔRL and ΔRL%, respectively) were calculated. Statistical analyses were conducted, including Mann-Whitney U tests, Spearman correlation analysis, Generalized Linear Mixed Model and multiple linear regression, to identify the associated predictors of reduced PDLA in maxillary incisors from risk factors including gender, age, U1-NA angle, Angle’s classification, sagittal skeletal pattern (ANB angle), vertical skeletal pattern, history of chronic periodontitis, tooth extraction status, and treatment duration.

**Results:**

At the end of active orthodontic treatment, ΔPDLA and ΔPDLA% were significantly higher in the following subgroups compared with the reference group: patients with ANB angle > 3° [Maxillary right central incisor (T11) ΔPDLA: 9.49(13.37) mm, Maxillary left central incisor (T21) ΔPDLA: 11.02(14.45) mm, *P* < 0.05; ΔPDLA%: T11 6.73(8.20) %, T21 7.05(8.66) %, *P* < 0.05], tooth extraction treatment group [T11 ΔPDLA: 11.83(14.14) mm, T21 ΔPDLA: 11.82(16.67) mm, *P* < 0.05; ΔPDLA%: T11 7.54 (10.13) %, T21 8.23(10.32) %, *P* = 0.058], and treatment duration > 2 years [T11 ΔPDLA: 9.34(14.83) mm, T21 ΔPDLA: 11.94(15.56) mm, *P* = 0.059; ΔPDLA%: T11 7.00 (10.87) %, T21 8.37(8.73) %, *P* < 0.05]. Additionally, the U1-NA angle > 28.5° subgroup showed marginally significantly higher ΔPDLA [11.46(25.13) mm, *P* = 0.082], and patients with skeletal Class II malocclusion exhibited a marginally significantly higher ΔPDLA% [T11 7.53 (12.03) %, T21 6.91(9.24) %, *P* = 0.059] relative to controls. Significant predictors of root resorption included adult age [T11 ΔRL: 0.63(1.30) mm, T21 ΔRL: 0.98(1.33) mm, *P* < 0.05; T11 ΔRL%: 5.41(11.20) %, T21 ΔRL%: 8.16(10.20) %, *P* < 0.05], and tooth extraction treatment [T11 ΔRL: 0.73(1.42) mm, T21 ΔRL: 1.12(1.41) mm, *P* < 0.01; T11 ΔRL%: 6.33(12.03) %, T21 ΔRL%: 8.20(11.84) %, *P* < 0.05]. Multiple linear regression revealed that ΔRL%, as a post-orthodontic outcome variable, was independently positively associated with ΔPDLA% (β = 0.536, *P* < 0.001). Among patient-related and treatment-related factors, the pre-treatment U1-NA angle significantly positively affected ΔPDLA% (β = 0.257, *P* < 0.01). Pre-treatment ∠ANB (*P* = 0.055) and treatment duration (*P* = 0.082) demonstrated marginal statistical significance, while age and tooth extraction status exerted no significant effects (all *P* > 0.05).

**Conclusion:**

Subgroup analyses identified adulthood, pre‑treatment ∠ANB > 3°, pre‑treatment U1‑NA angle > 28.5°, tooth extraction, and treatment duration > 2 years as associated risk factors for supporting tissue loss around the teeth. Multiple linear regression further confirmed the independent effects of pre‑treatment U1‑NA angle, ∠ANB, and treatment duration on periodontal ligament area reduction. A comprehensive pre‑treatment evaluation of combined skeletal‑dental features is critical for individualized orthodontic risk stratification and clinical decision‑making.

## Introduction

Orthodontic treatment, as a pivotal medical intervention for reconstructing occlusal function and enhancing facial aesthetics, has been globally popularized. However, Orthodontically Induced Inflammatory Root Resorption (OIRR), one of the most prevalent iatrogenic complications, remains a persistent clinical dilemma for both practitioners and patients. Although the majority of cases exhibit mild root resorption with no discernible clinical significance, a subset may develop severe root shortening, accompanied by tooth mobility or even loss. Among all dentitions, maxillary central incisors are the most frequently affected site for OIRR, typically manifesting more severe resorption [1–4]. This phenomenon is primarily attributed to their unique anatomical position, distinctive root morphology, as well as the magnitude and pattern of tooth movement during orthodontic therapy. Therefore, in-depth research on the safety of maxillary central incisors in orthodontic treatment has profound clinical guidance significance.

The periodontal ligament (PDL) is a dense connective tissue that surrounds the tooth root and connects the root to the inner wall of the alveolar bone. However, due to technical limitations, cone-beam computed tomography (CBCT) cannot identify the alveolar crest fibers within it [5]. In addition, given that the voxel size of CBCT is 0.3 mm, it fails to reflect periodontal ligament spaces thinner than 0.3 mm. Therefore, the periodontal ligament area (PDLA) measured in this study is different from the biological PDL; it refers to the three-dimensional surface area of the periodontal ligament space between the root surface and the inner wall of the alveolar bone [6]. Tooth stability depends not only on root length (RL), but also on alveolar bone height and the presence of dehiscence or fenestration. Diminished alveolar bone height, root resorption, and the formation of dehiscence or fenestration contribute to a reduction in PDLA. Thus, PDLA measurement offers a more comprehensive assessment of dental health. Furthermore, it does not rely on standard coordinate systems and is unimpaired by positional changes caused by tooth inclination, rotation, and other such factors. However, traditional manual tracking and measurement techniques are time-consuming and labor-intensive, severely limiting the practicality of PDLA in clinical practice.

With the rise of artificial intelligence technology, medical image segmentation algorithms based on deep learning have provided revolutionary solutions to this problem. Specifically, Convolutional Neural Networks (CNNs) represented by U-Net, SegResNet, etc. have been proven to achieve high-precision and efficient automatic segmentation of complex anatomical structures such as teeth and jawbones in CBCT images [7, 8]. Our researches have previously developed a deep learning-based automated periodontal ligament segmentation system for CBCT images, and its reliability in measuring the PDLA of maxillary central incisors has been validated [6, 9], which makes it possible to perform fine quantitative analysis of periodontal structure on large sample CBCT data. On this basis, this study will investigate the correlation between individual patient factors and treatment factors with changes in PDLA and root resorption. It is anticipated that associated risk factors for adverse outcomes of maxillary central incisors can be identified through statistical analysis, and a risk-prediction model may be established to provide a reference basis for orthodontic clinicians in pretreatment risk evaluation and dynamic intra-treatment monitoring. This approach aims to achieve optimal orthodontic outcomes while maximizing the preservation of periodontal tissue health and stability.

## Materials and methods

This retrospective case-control study was approved by the Bioethics Committee of Beijing Friendship Hospital, Capital Medical University (Approval No. 2025-P2-607-01) and conducted in strict accordance with the Declaration of Helsinki [10]. Routine clinical data were used in this study. Verbal informed consent was obtained via telephone from all contactable patients, and a waiver of informed consent was granted by the Bioethics Committee of Beijing Friendship Hospital, Capital Medical University for those who were unreachable.

Sample size was determined using Power Analysis and Sample Size Software (PASS 21.0, NCSS, LLC, Kaysville, UT, USA). Based on the findings of the present study’s pilot trial (PDLA measurements in 20 maxillary central incisors from 10 patients before and after orthodontic treatment), with a significance level (α) of 0.05 and a statistical power (1-β) of 80%, a minimum of 26 maxillary central incisors from 13 patients was calculated to be sufficient for the significance analysis of measurement variables. For the multiple linear regression analysis, which included 6 independent variables, the minimum sample size was set at 60 subjects in accordance with the 10 Subjects Per Variable rule to ensure statistical validity.

This study retrospectively retrieved CBCT datasets from patients who had completed comprehensive orthodontic treatment in the Department of Stomatology, Beijing Friendship Hospital, Capital Medical University, between May 2021 and December 2022, from the hospital’s dedicated CBCT imaging database. All CBCT images were acquired using a single CBCT scanner (NewTom CT, Cefla QR Verona, Verona, Italy) under standardized acquisition parameters: 110 kV tube voltage, 2.1–4.4 mA tube current, 18 × 16 cm field of view, 0.3 mm isotropic voxel size, 360° rotation, and 3.6 s exposure time [6, 11].

### Inclusion criteria

Patients were included if they met all the following criteria:

1. Underwent monotherapy with straight-wire orthodontic appliances with a 0.022 × 0.028-inch bracket slot size; the archwire sequence followed standard clinical protocols, with space closure and anterior segment retraction performed exclusively using a 0.018 × 0.025-inch stainless steel rectangular archwire.
2. No age or gender restrictions at the initial orthodontic consultation; all maxillary central incisors demonstrated complete root development with fully closed apical foramina.
3. Underwent CBCT imaging before treatment initiation and at the completion of active treatment, with the scanning range spanning from the supraorbital margin superiorly to the inferior border of the chin inferiorly.

### Exclusion criteria

Cases were excluded if any of the following conditions were present:

1. The target maxillary central incisors had pathological conditions, including post-traumatic discoloration, a history of root canal therapy or core crown restoration, chronic periapical periodontitis, or severe pre-existing root resorption.
2. Patients with chronic periodontitis who have not received standard periodontal basic treatment or periodontal treatment for less than 3 months, with bleeding on probing sites ≥ 30% or periodontal probing depth >6 mm.
3. CBCT images of insufficient quality for quantitative analysis (e.g., blurring or artifacts caused by head rotation, translation, or motion).

A total of 63 patients were ultimately included in the final analysis, with 14 males and 49 females aged 12–43 years. Both the left and right maxillary central incisors were measured in each patient.

Retrospective data extraction and cephalometric analysis were performed to collect the following variables: (1) Demographic characteristics (gender, age); (2) Pre-treatment U1-NA angle; (3) Angle’s classification; (4) Sagittal skeletal pattern (ANB angle, ∠ANB); (5) Vertical skeletal pattern (Frankfort Mandibular Plane Angle, FMA); (6) History of chronic periodontitis; (7) Extraction status (extracted or not); (8) Treatment duration.

**Fig. 1:**
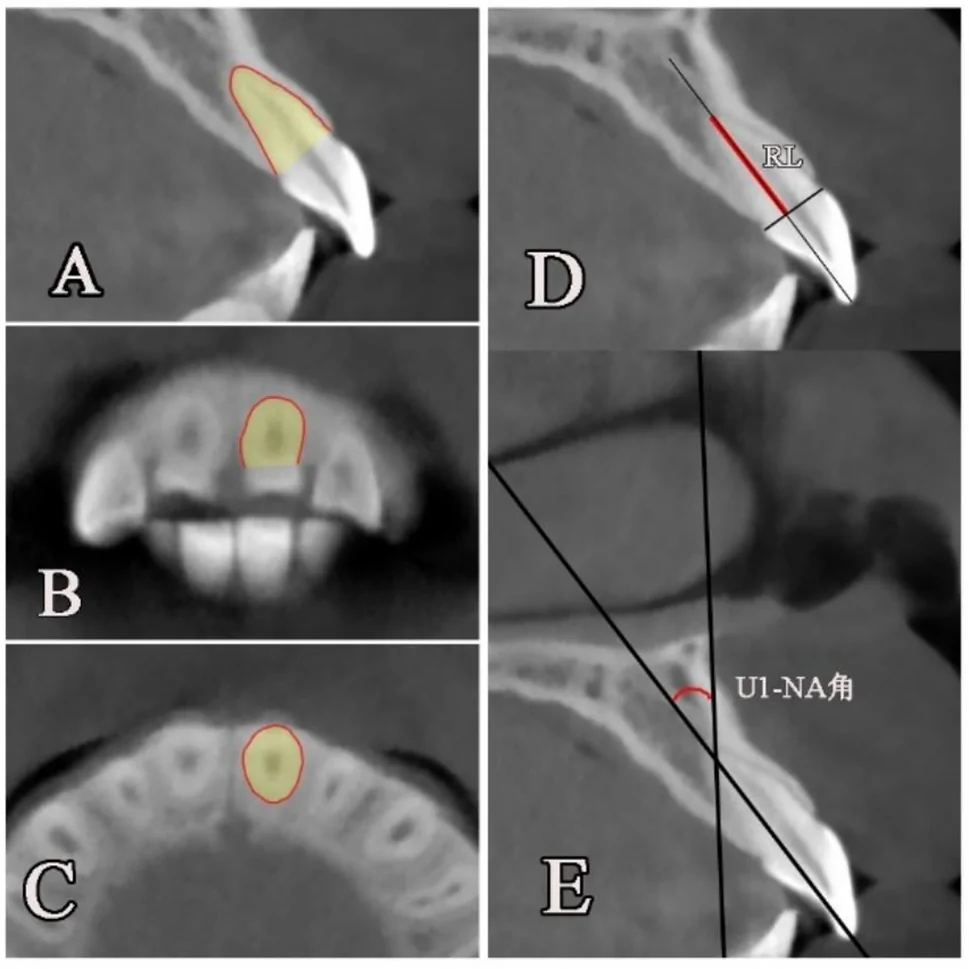
**A** The periodontal ligament (PDL) marked on the sagittal plane, i.e., the region where the root overlaps with the surrounding alveolar bone (The yellow area represents the root, and the red line is a schematic diagram of PDL segmentation.); **B** PDL marked on the coronal plane; **C** PDL marked on the transverse plane; **D** Root Length (RL): the distance from the intersection of the long axis of the maxillary central incisor and the cementoenamel junction to the tooth apex (the red line); **E** U1-NA Angle: the angle between the long axis of the maxillary central incisor and the line connecting nasion (N) to alveolar point (A), measured on the sagittal plane

PDLA Measurement: The PDL was defined as the three-dimensional region where the root overlaps with the surrounding alveolar bone (Fig. 1A, B and C). PDLA was measured at pre-treatment (PDLA₀) and post-treatment (PDLA₁). The absolute reduction in PDLA (ΔPDLA) was calculated as PDLA₀ − PDLA₁, and the percentage reduction (ΔPDLA%) was computed as $\left(\triangle\mathrm{PDLA}\;/\;\mathrm{PDLA}_0\right)\times100\%$.

RL and U1-NA Angle Measurement: The Dolphin Imaging 3D program ver. 11.9 (Dolphin Imaging and Management Solutions, Chatsworth, CA, America) was used for image reproduction on the computer display. During head positioning, the Frankfort horizontal plane was taken as the horizontal plane, and a vertical line drawn through the midpoint of the bilateral infraorbital margins formed a sagittal plane with the sella turcica point. The following measurements were taken under the Digitize/Measurement menu:

1. RL: RL was defined as the distance from the intersection of the maxillary central incisor’s long axis and the cementoenamel junction to the tooth apex, measured on the sagittal plane through the long axis of the maxillary central incisors (Fig. 1D). RL was recorded at pre-treatment (RL₀) and post-treatment (RL₁). The absolute reduction in RL (ΔRL) was calculated as RL₀ − RL₁, and the percentage reduction (ΔRL%) was computed as $\left(\triangle\mathrm{RL}\;/\;\mathrm{RL}_0\right)\times100\%$.
2. U1-NA Angle: This angle was defined as the angle between the maxillary central incisor’s long axis and the line connecting nasion (N) to alveolar point (A), measured on the sagittal plane through the tooth’s long axis (Fig. 1E).

All manual measurements were performed by two attending orthodontists with ≥ 5 years of clinical experience. Each examiner conducted duplicate measurements of all variables with a 2-week interval. Intra-examiner consistency was assessed using paired t-tests (two-sided *α* = 0.05). Inter-examiner reliability was evaluated by calculating the intraclass correlation coefficient (ICC) based on the mean values of the two examiners; an ICC > 0.85 was defined as excellent agreement.

Statistical analyses were performed using SPSS Statistics, version 27.0 (IBM, Armonk, NY, USA). Normality of data distribution was assessed via the Shapiro-Wilk test (*α* = 0.05). Normally distributed data were presented as mean ± standard deviation (Mean ± SD), while non-normally distributed data were reported as median (interquartile range) [M (IQR)]. For normally distributed data, homogeneity of variance was tested using Levene’s test (*α* = 0.05). Between-group comparisons were conducted using independent-samples t-tests for normally distributed data with homogeneous variance, and Mann-Whitney U tests for non-normally distributed data (*α* = 0.05). Correlation analysis was performed using Pearson’s correlation for normally distributed data and Spearman’s correlation for non-normally distributed data. A positive correlation coefficient (*r*) indicated a positive association, while a negative *r* indicated a negative association (|r|: 0.0–0.2, very weak; 0.2–0.4, weak; 0.4–0.6, moderate; 0.6–0.8, strong; 0.8–1.0, very strong). Generalized linear mixed models (GLMMs) were employed to assess the effects of various risk factors. Patient ID was included as a random effect and tooth position as a repeated-measure variable to account for intra-patient correlation between bilateral maxillary central incisors. Multiple linear regression analysis was conducted with ΔPDLA% as the dependent variable, and age, pre-treatment ∠ANB, pre-treatment U1-NA angle, treatment duration, and ΔRL% as independent variables, using the enter method. In all statistical tests, *P* < 0.05 was considered statistically significant, and *P* < 0.01 was considered highly statistically significant.

## Results

### Reliability and normality tests

Paired *t*-tests were performed to assess intra- and inter-examiner consistency. No statistically significant differences were observed between duplicate measurements of all indicators by a single attending orthodontist, nor between the mean values of measurements from the two orthodontists (*P* > 0.05).

The ICC values for all measurement indicators ranged from 0.88 to 0.95, both between duplicate measurements by the same examiner (intra-examiner ICC: 0.89–0.94) and between the mean values of measurements from the two attending orthodontists (inter-examiner ICC: 0.88–0.95). These results confirmed excellent inter- and intra-examiner reliability.

Variables with a normal distribution (all Shapiro-Wilk *P* > 0.05) included PDLA_0_, PDLA_1_, RL_0_ and RL_1_, which are presented as Mean ± Standard Deviation (Mean ± SD). Variables with a non-normal distribution included ΔPDLA, ΔPDLA%, ΔRL and ΔRL%—key indicators reflecting periodontal support loss and root resorption—as well as age, ANB angle, treatment duration, and pre-treatment U1-NA angle. These non-normally distributed variables are presented as Median (Interquartile Range) [M (IQR)].

### Analysis of risk factors associated with PDLA reduction

#### Patient-related factors

##### Gender

A total of 63 patients were included in the analysis, consisting of 14 males and 49 females, with 126 maxillary central incisors measured bilaterally. No statistically significant differences in ΔPDLA, ΔPDLA%, ΔRL, and ΔRL% were observed between males and females (all *P* > 0.05). These results suggest that gender may not be a clinically relevant risk factor for PDLA reduction in maxillary central incisors after orthodontic treatment (Table 1).

**Table 1:** PDLA and RL Differences by Gender

	Tooth position	Gender		*F*	*P*
		Male (*n* = 14)	Female (*n* = 49)		
△RL (mm)	T11	0.46 (0.84)	0.60 (0.94)	0.134	0.715
	T21	0.92 (1.00)	0.81 (1.14)		
△RL%	T11	3.58 (8.66)	5.39 (8.29)	0.110	0.740
	T21	8.91 (9.17)	6.17 (8.82)		
△PDLA (mm^2^)	T11	8.59 (8.04)	7.95 (13.43)	0.044	0.834
	T21	8.97 (11.55)	9.28 (15.52)		
△PDLA%	T11	5.39 (6.33)	4.89 (8.43)	0.079	0.778
	T21	6.33 (7.11)	6.35 (10.09)		

Data are presented as median (interquartile range). Generalized linear mixed models (GLMMs) were employed to assess gender-based differences. ΔRL, change in root length; ΔRL%, percentage change in root length; ΔPDLA, change in periodontal ligament area; ΔPDLA%, percentage change in periodontal ligament area; T11, right maxillary central incisor; T21, left maxillary central incisor

##### Age

Spearman correlation analysis revealed a significant positive correlation between age and ΔRL (*r* = 0.199, *P* = 0.026) and ΔRL% (*r* = 0.194, *P* = 0.030).

Patients were stratified into two age groups: 12–18 years and 19–43 years. The 12–18 years group exhibited lower values of ΔRL, ΔRL%, ΔPDLA and ΔPDLA% compared with the 19–43 years group. Specifically, the differences in ΔRL and ΔRL% between the two groups were statistically significant (*P* < 0.05) (Table 2).

**Table 2:** PDLA and RL differences among age groups

	Tooth position	Age(years)		*F*	*P*
		12–18 (*n* = 20)	19–43 (*n* = 43)		
△RL (mm)	T11	0.53 (0.58)	0.63 (1.30)	4.984	0.027*
	T21	0.59 (0.64)	0.98 (1.33)		
△RL%	T11	4.57 (5.41)	5.41 (11.20)	4.311	0.040*
	T21	5.02 (7.35)	8.16 (10.20)		
△PDLA (mm^2^)	T11	7.68 (13.40)	9.29 (12.37)	0.043	0.835
	T21	8.13 (12.11)	8.75 (17.14)		
△PDLA%	T11	4.22 (8.81)	6.45 (7.84)	0.326	0.569
	T21	6.23 (8.64)	6.35 (11.07)		

Data are presented as median (interquartile range). GLMMs were employed to assess age-based differences; *: *P*<0.05

##### Initial inclination of maxillary central incisors

**Fig. 2:**
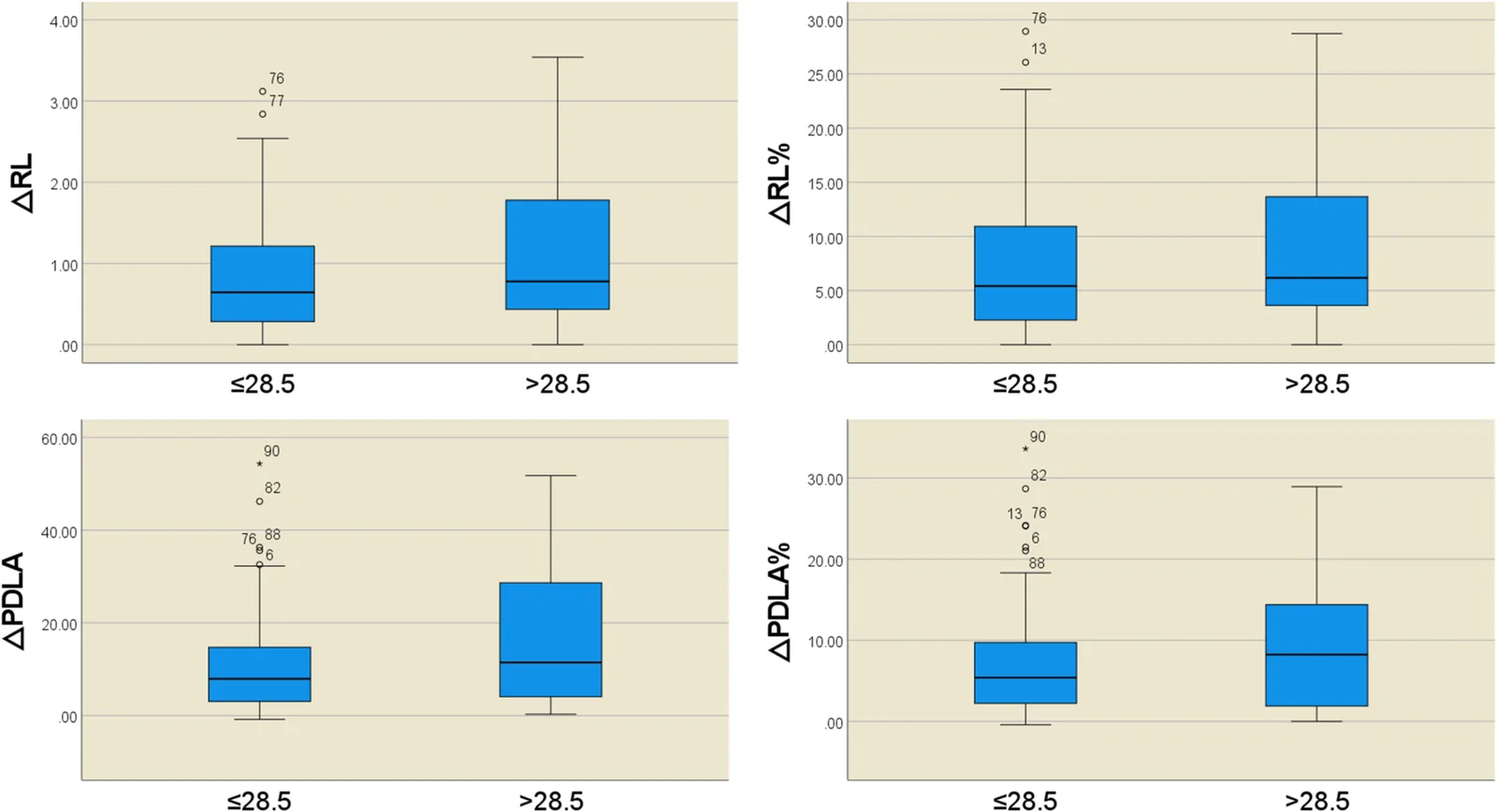
Distribution of variables stratified by initial inclination of maxillary central incisors

A total of 116 valid pre-treatment U1-NA angle measurements were included in the analysis, with values ranging from 1.00° to 38.30°. Based on the established reference value for the U1-NA angle in the permanent dentition of Chinese populations (22.8 ± 5.7°) (i.e., mean + 1 SD = 28.5°) [12], patients were categorized into non-protruded (≤ 28.5°) and protruded (> 28.5°) groups. The protruded group demonstrated higher ΔRL, ΔRL%, ΔPDLA, and ΔPDLA% compared to the non-protruded group. A marginally statistically significant difference in ΔPDLA was observed between the two groups (*P* = 0.082) (Table 3; Fig. 2).

**Table 3:** PDLA and RL Differences Between Protruded and Non-protruded Groups

	Initial inclination classification		Z	*P*
	Non-protruded (U1-NA ≤ 28.5º) (*n* = 88)	Protruded (U1-NA > 28.5º) (*n* = 28)		
△RL (mm)	0.65 (0.94)	0.78 (1.40)	-1.074	0.283
△RL%	5.43 (8.68)	6.18 (10.72)	-0.829	0.407
△PDLA (mm^2^)	7.93 (11.85)	11.46 (25.13)	-1.742	0.082
△PDLA%	5.41 (7.64)	8.23 (13.09)	-1.239	0.215

Data are presented as median (interquartile range). GLMMs were employed to assess differences in PDLA and RL Between Protruded and Non-protruded Groups

##### Angle’s classification

The study cohort included 36 patients with Class I malocclusion, 21 patients with Class II malocclusion and 6 patients with Class Ⅲ malocclusion. The Class III malocclusion was excluded from further statistical analysis due to its small sample size (*n* = 6), which may have reduced statistical power. Numerically, patients with Class II malocclusion presented higher ΔRL, ΔRL%, ΔPDLA, and ΔPDLA% than those with Class I malocclusion, yet none of these differences reached statistical significance (all *P* > 0.05). (Table 4). Consistent with the Angle classification, the sagittal skeletal pattern, which reflects the underlying jaw relationship, was further analyzed to explore its impact on PDLA and RL changes.

**Table 4:** PDLA and RL Differences Between Angle Class I and II Patients

	Tooth position	Angle’s classification		*F*	*P*
		I (*n* = 36)	II (*n* = 21)		
△RL (mm)	T11	0.58 (1.11)	0.60 (0.94)	0.011	0.918
	T21	0.71 (1.12)	0.88 (1.23)		
△RL%	T11	5.25 (8.09)	5.41 (8.95)	0.284	0.595
	T21	5.33 (9.06)	8.09 (11.22)		
△PDLA (mm^2^)	T11	7.68 (12.86)	9.31 (11.70)	0.939	0.335
	T21	7.37 (16.45)	11.05 (12.46)		
△PDLA%	T11	4.48 (8.55)	6.49 (8.13)	2.004	0.160
	T21	4.96 (11.12)	7.35 (6.80)		

Data are presented as median (interquartile range). GLMMs were employed to assess differences

##### Sagittal skeletal pattern

Based on the standard reference value of ∠ANB (2.7° ± 2.0°) for the permanent dentition in Chinese populations [12], patients were classified into skeletal Class I (0°< ANB ≤ 5°, *n* = 36), skeletal Class II (ANB > 5°, *n* = 21), and skeletal Class III (ANB ≤ 0°, *n* = 6). The skeletal Class III group was excluded from further statistical analysis due to its small sample size (*n* = 6). Patients with skeletal Class II exhibited higher ΔPDLA, ΔPDLA%, ΔRL, and ΔRL% than those with skeletal Class I. A near-statistically significant difference was observed in ΔPDLA% between the two groups (*P* = 0.059). (Table 5).

**Table 5:** PDLA and RL Differences Between Skeletal Class I and II Patients

	Tooth position	Skeletal classification		*F*	*P*
		I (*n* = 36) (0°<∠ANB ≤ 5°)	II (*n* = 21) (∠ANB > 5°)		
△RL (mm)	T11	0.53 (1.22)	0.72 (0.81)	0.534	0.467
	T21	0.52 (0.90)	1.06 (1.19)		
△RL%	T11	4.25 (8.82)	6.62 (8.42)	1.706	0.194
	T21	4.83 (7.80)	10.49 (10.24)		
△PDLA (mm^2^)	T11	6.96 (10.57)	10.61 (16.72)	2.374	0.126
	T21	8.33 (12.68)	10.14 (15.01)		
△PDLA%	T11	4.48 (6.39)	7.53 (12.03)	3.626	0.059
	T21	5.39 (9.35)	6.91 (9.24)		

Data are presented as median (interquartile range). GLMMs were employed to assess differences

**Fig. 3:**
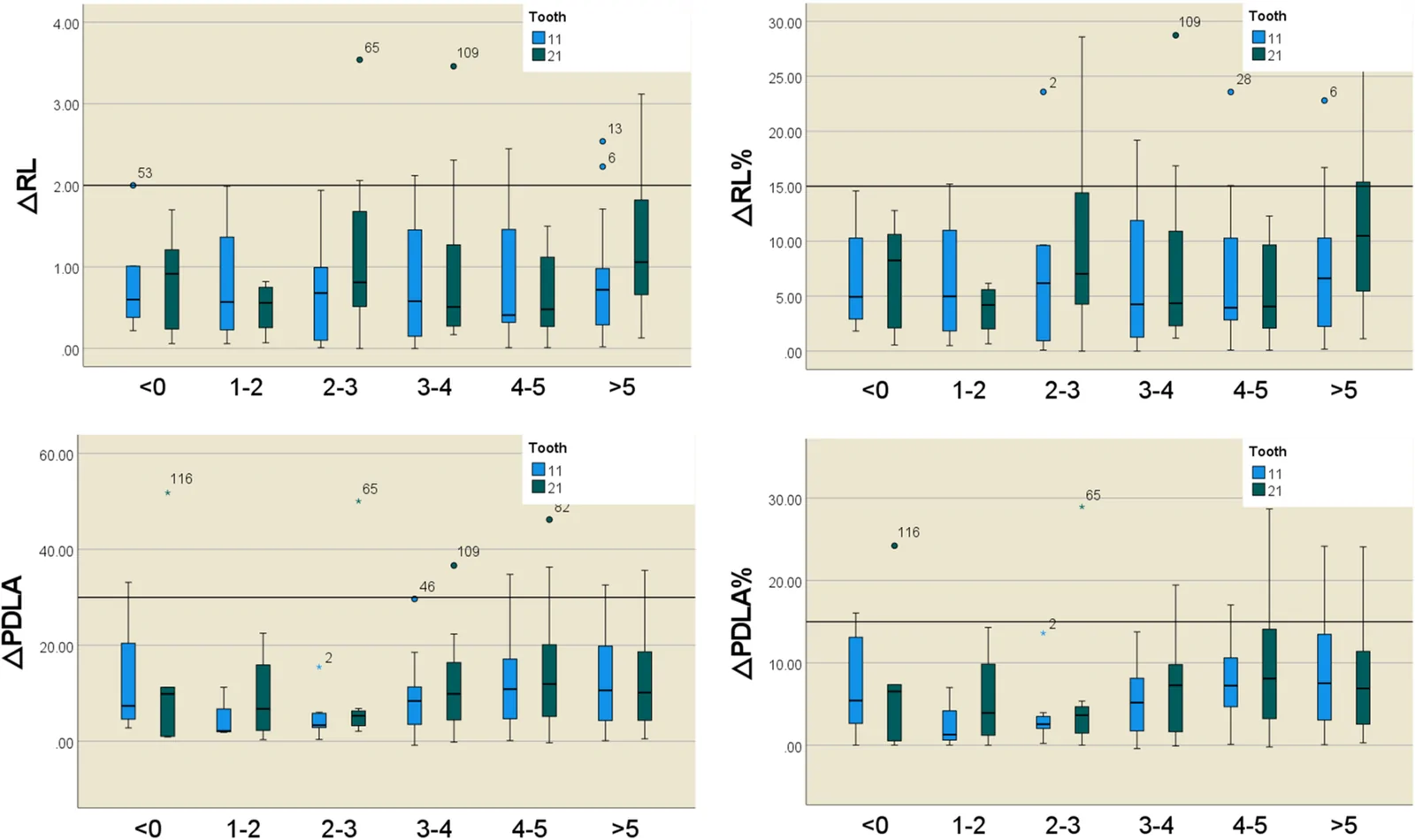
Distribution of △RL, △RL%, △PDLA and △PDLA% after subdividing ∠ANB. Lines on the Y-axis represent the median of each group. T11, right maxillary central incisor; T21, left maxillary central incisor

Spearman correlation analysis demonstrated a significant positive correlation between ∠ANB and ΔPDLA (*r* = 0.211*, *P* = 0.018) and ΔPDLA% (*r* = 0.246**, *P* = 0.006). When ∠ANB was subdivided into seven categories (< 0°, 0–1°, 1.1–2°, 2.1–3°, 3.1–4°, 4.1–5°, > 5°), ΔPDLA and ΔPDLA% were found to be smaller in the 0°<∠ANB < 3° subgroup, with no statistically significant differences in ΔRL, ΔRL%, ΔPDLA, or ΔPDLA% among all subgroups (*P* > 0.05) (Fig. 3).

**Fig. 4:**
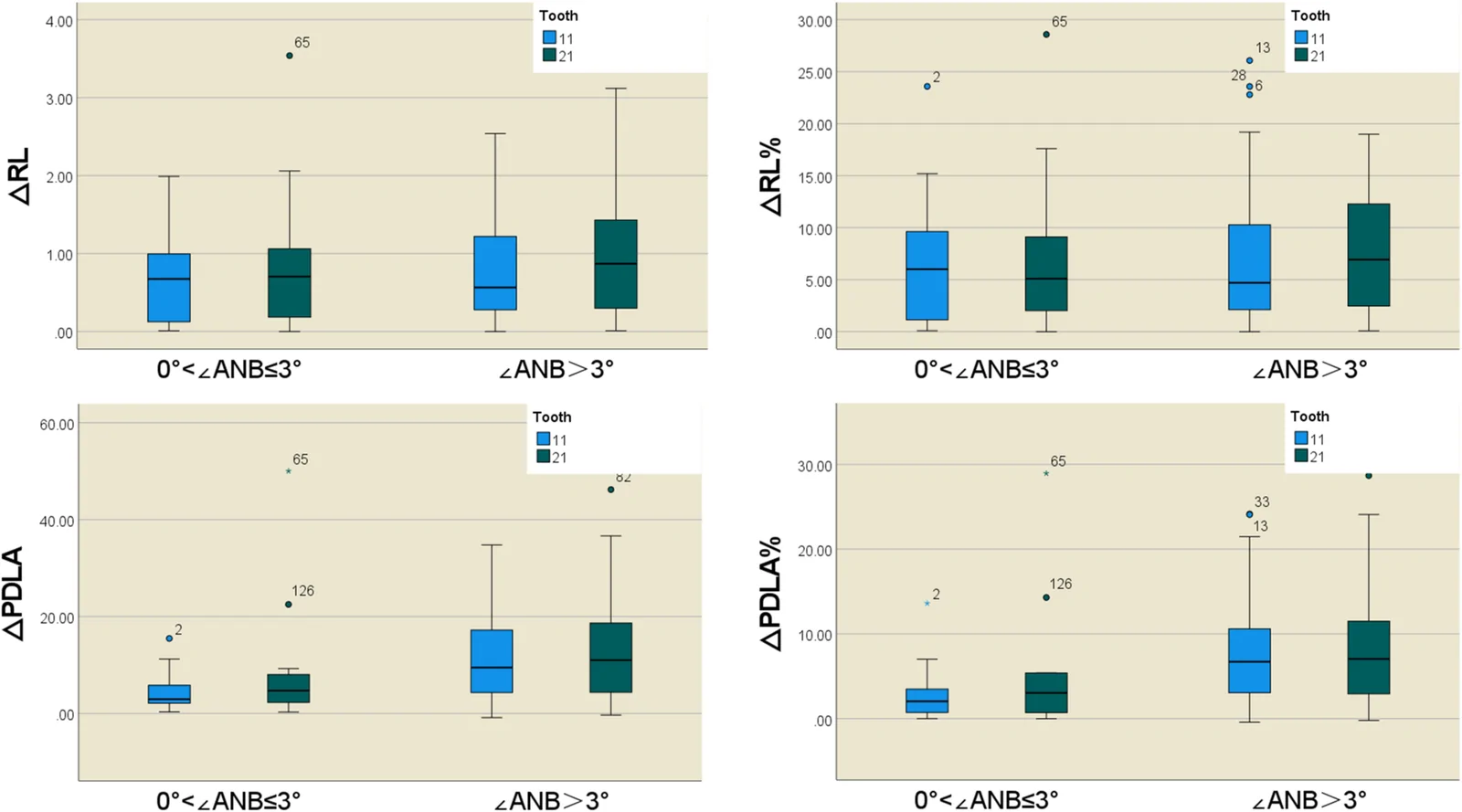
Distribution of variables across different ∠ANB groups

Subsequently, patients were reclassified into two groups based on ∠ANB: 0°<∠ANB≤ 3° and ∠ANB > 3°. The 0°<∠ANB≤ 3° group exhibited significantly lower ΔPDLA and ΔPDLA% compared with the ∠ANB > 3° group (*P* < 0.05) (Table 6; Fig. 4).

**Table 6:** RL and PDLA Differences Among ∠ANB Groups

	Tooth position	∠ANB classification		*F*	*P*
		0°<∠ANB ≤ 3° (*n* = 12)	∠ANB>3° (*n* = 46)		
△RL (mm)	T11	0.68 (0.92)	0.57 (0.99)	0.144	0.706
	T21	0.71 (1.05)	0.87 (1.15)		
△RL%	T11	6.01 (8.82)	4.71 (8.39)	0.072	0.789
	T21	5.10 (8.81)	6.93 (10.00)		
△PDLA (mm^2^)	T11	2.96 (3.84)	9.49 (13.37)	5.786	0.019*
	T21	4.75 (6.48)	11.02 (14.45)		
△PDLA%	T11	2.05 (3.25)	6.73 (8.20)	5.399	0.024*
	T21	3.04 (5.03)	7.05 (8.66)		

Data are presented as median (interquartile range). GLMMs were employed to assess differences. *: *P*<0.05

##### Vertical skeletal pattern

Vertical skeletal patterns were classified based on FMA into three types: average-mandible-angle type (22°≤FMA ≤ 32°, *n* = 35), low-mandible-angle type (FMA < 22°, *n* = 3), and high-mandible-angle type (FMA > 32°, *n* = 25) [12]. Similar to the skeletal Class III group, the low-mandible-angle type was excluded from statistical analysis due to its small sample size (*n* = 3). The average-mandible-angle type presented higher ΔRL and ΔRL% but lower ΔPDLA and ΔPDLA% compared with the high-mandible-angle type; however, none of these differences were statistically significant (*P* > 0.05) (Table 7).

**Table 7:** PDLA and RL Differences by Vertical Skeletal Pattern

	Tooth position	Vertical skeletal pattern classification		*F*	*P*
		average-mandible-angle (22°≤FMA ≤ 32°) (*n* = 35)	high-mandible-angle (FMA > 32°) (*n* = 25)		
△RL (mm)	T11	0.67 (1.00)	0.53 (0.84)	0.007	0.934
	T21	0.85 (1.00)	0.82 (1.40)		
△RL%	T11	5.83 (9.08)	4.19 (6.92)	0.053	0.819
	T21	7.03 (8.90)	6.30 (11.59)		
△PDLA (mm^2^)	T11	6.61 (14.02)	10.61 (13.49)	0.841	0.363
	T21	8.10 (12.98)	11.70 (29.13)		
△PDLA%	T11	4.29 (7.77)	6.49 (9.87)	0.457	0.502
	T21	6.35 (8.19)	7.18 (16.38)		

Data are presented as median (interquartile range). GLMMs were employed to assess differences

##### Chronic periodontitis

A total of 7 patients with chronic periodontitis were included in the study, all of whom received comprehensive periodontal therapy to control periodontal inflammation prior to the initiation of orthodontic treatment. Statistical analysis showed that patients with chronic periodontitis exhibited smaller ΔRL, ΔRL%, ΔPDLA, and ΔPDLA% compared with those without chronic periodontitis, but these differences were not statistically significant (*P* > 0.05) (Table 8).

**Table 8:** PDLA and RL Differences by Chronic Periodontitis

	Tooth position	Chronic Periodontitis		*F*	*P*
		None (*n* = 56)	Yes (*n* = 7)		
△RL (mm)	T11	0.65 (0.90)	0.32 (0.38)	0.578	0.450
	T21	0.84 (1.03)	0.66 (1.67)		
△RL%	T11	5.62 (8.14)	2.84 (3.17)	0.057	0.812
	T21	6.48 (8.78)	5.47 (13.26)		
△PDLA (mm^2^)	T11	8.43 (14.19)	5.45 (11.04)	0.662	0.419
	T21	9.71 (14.69)	6.79 (11.98)		
△PDLA%	T11	5.49 (8.47)	3.84 (7.70)	0.239	0.627
	T21	6.69 (9.67)	5.45 (7.64)		

Data are presented as median (interquartile range). GLMMs were employed to assess differences

#### Treatment-related factors

##### Extraction therapy

**Fig. 5:**
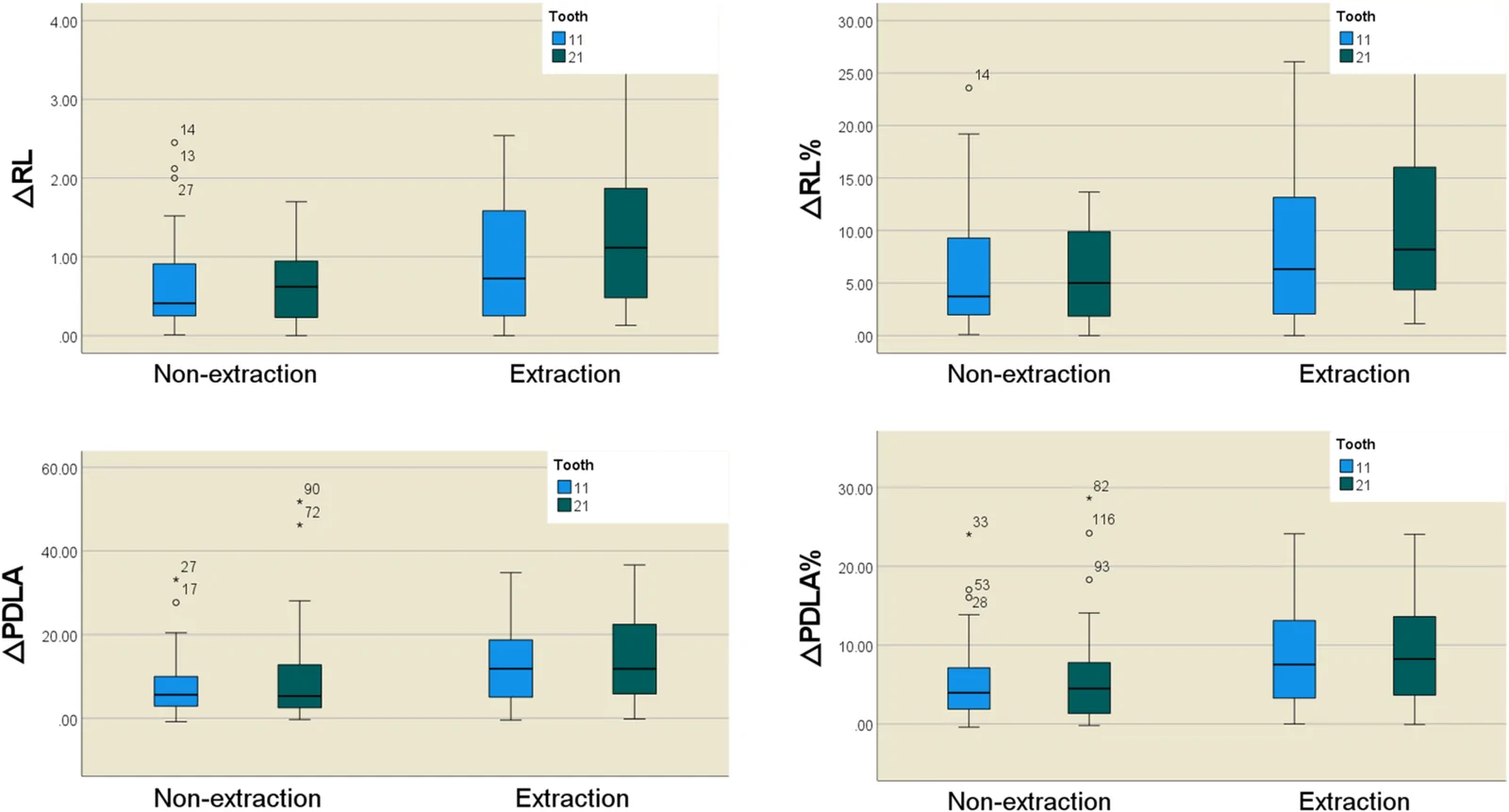
Box-and-whisker plots presenting the distributions of ΔRL, ΔRL%, ΔPDLA, and ΔPDLA% in maxillary central incisors (teeth 11 and 21) across extraction and non-extraction groups

Among all 63 enrolled subjects, 32 patients received premolar extraction. The extraction group showed significantly greater ΔRL, ΔRL%, and ΔPDLA compared with those in the non-extraction group (*P* < 0.05), whereas ΔPDLA% showed a borderline significant difference between groups (*P* = 0.058) (Table 9; Fig. 5).

**Table 9:** PDLA and RL Differences by Extraction Treatment Group

	Tooth position	Extraction group		*F*	*P*
		None (*n* = 31)	Yes (*n* = 32)		
△RL (mm)	T11	0.41 (0.74)	0.73 (1.42)	7.215	0.009**
	T21	0.62 (0.76)	1.12 (1.41)		
△RL%	T11	3.73 (7.82)	6.33 (12.03)	6.472	0.014*
	T21	5.01 (8.35)	8.20 (11.84)		
△PDLA (mm^2^)	T11	5.62 (7.81)	11.83 (14.14)	4.743	0.033*
	T21	5.29 (12.18)	11.82 (16.67)		
△PDLA%	T11	3.96 (5.44)	7.54 (10.13)	3.729	0.058
	T21	4.48 (6.94)	8.23 (10.32)		

Data are presented as median (interquartile range). GLMMs were employed to assess differences. *: *P*<0.05; **: *P*<0.01

##### Treatment duration

**Fig. 6:**
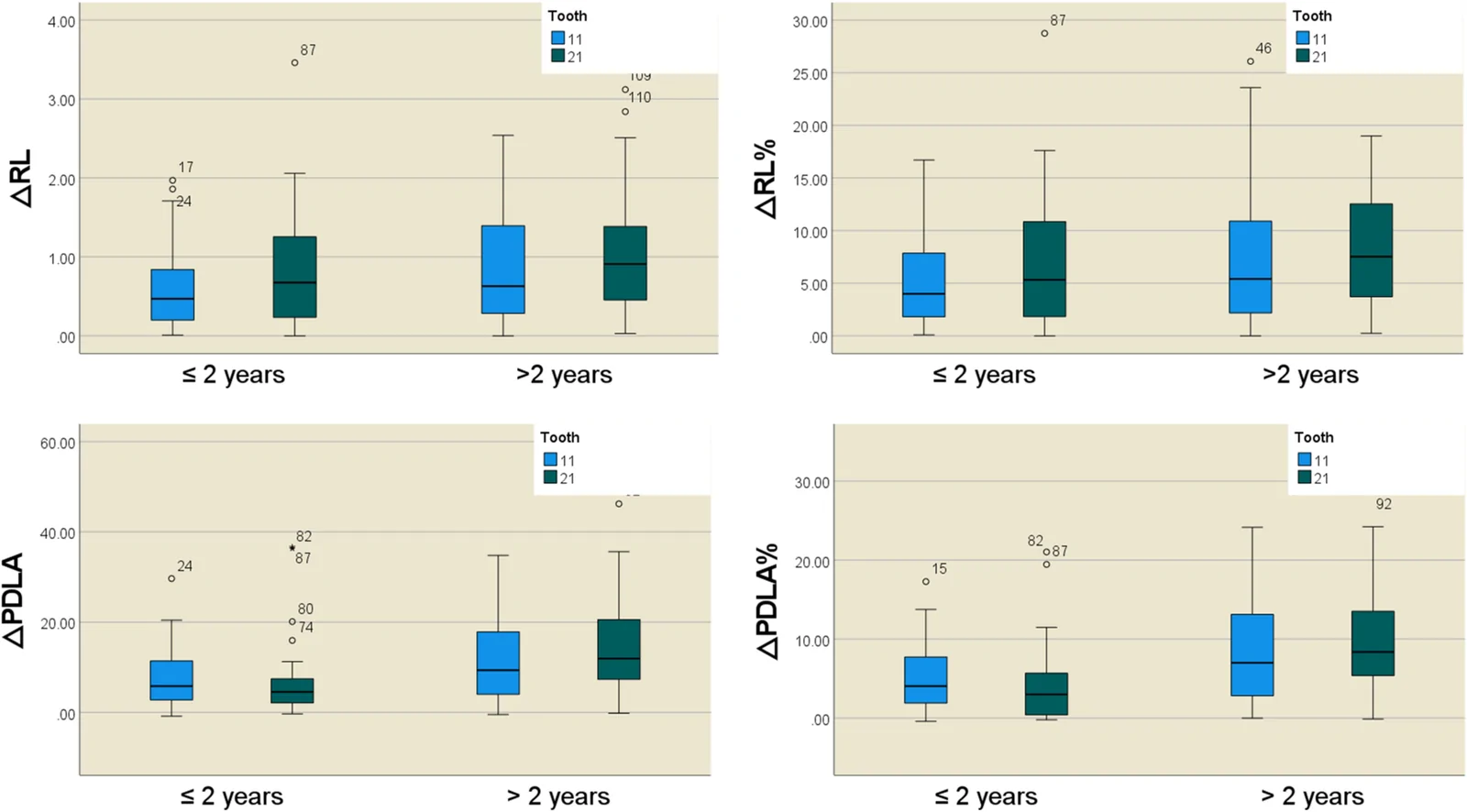
Distribution of △RL, △RL%, △PDLA and △PDLA% grouped by treatment duration.

Patients were divided into two groups based on treatment duration (defined as the period from bracket bonding to debonding): >2 years and ≤ 2 years. Patients with treatment duration > 2 years exhibited higher ΔRL and ΔRL% than those with treatment duration ≤ 2 years, but these differences were not statistically significant (*P* > 0.05). Additionally, ΔPDLA was slightly increased in the > 2 years group compared with the ≤ 2 years group, though this difference was marginally significant (*P* = 0.059). The difference in ΔPDLA% between the two groups was statistically significant (*P* < 0.05) (Table 10; Fig. 6).

**Table 10:** PDLA and RL Differences by Treatment Duration

	Tooth position	Treatment Duration(years)		*F*	*P*
		≤ 2 (*n* = 24)	>2 (*n* = 39)		
△RL (mm)	T11	0.47 (0.65)	0.63 (1.18)	2.122	0.150
	T21	0.68 (1.05)	0.91 (0.99)		
△RL%	T11	4.00 (6.60)	5.41 (9.38)	1.789	0.186
	T21	5.33 (9.31)	7.53 (9.41)		
△PDLA (mm^2^)	T11	5.83 (9.07)	9.34 (14.83)	3.691	0.059
	T21	4.56 (5.67)	11.94 (15.56)		
△PDLA%	T11	4.05 (6.10)	7.00 (10.87)	6.205	0.014*
	T21	3.00 (5.94)	8.37(8.73)		

Data are presented as median (interquartile range). GLMMs were employed to assess differences. *: *P*<0.05

### Multiple linear regression analysis

To further explore baseline pre-treatment risk factors for ΔPDLA% and evaluate the independent association between ΔRL% and ΔPDLA%, multiple linear regression analysis using the enter method was performed. Bilateral maxillary central incisors (teeth 11 and 21) were included, with tooth side (right vs. left) incorporated as a covariate to adjust for intra-individual correlation between paired-tooth data. Other covariates included age, pre-treatment ∠ANB, pre-treatment U1-NA angle, treatment duration, and tooth extraction status.

The initial regression model including 116 tooth-level observations was statistically significant overall (*F* = 9.521, *P* < 0.001), with an adjusted *R²* of 0.342, explaining 34.2% of the variance in ΔPDLA%. The Durbin-Watson statistic was 2.160, indicating no autocorrelation of residuals. Collinearity diagnostics confirmed the absence of severe multicollinearity, with the variance inflation factor (VIF) for all independent variables less than 2. Regression results showed that ΔRL% was significantly positively associated with ΔPDLA% (B = 0.523, standardized *β* = 0.502, *t* = 5.935, *P* < 0.001). The pre-treatment U1-NA angle was also significantly positively associated with ΔPDLA% (B = 0.186, standardized *β* = 0.196, *t* = 2.143, *P* < 0.05). The pre-treatment ∠ANB (*P* = 0.052) and treatment duration (*P* = 0.096) approached statistical significance, while age, tooth extraction status or tooth position displayed significant independent predictive effects on ΔPDLA% (all *P* > 0.05). Two extreme outliers with standardized residuals > 3 were identified, which might compromise model stability.

**Fig. 7:**
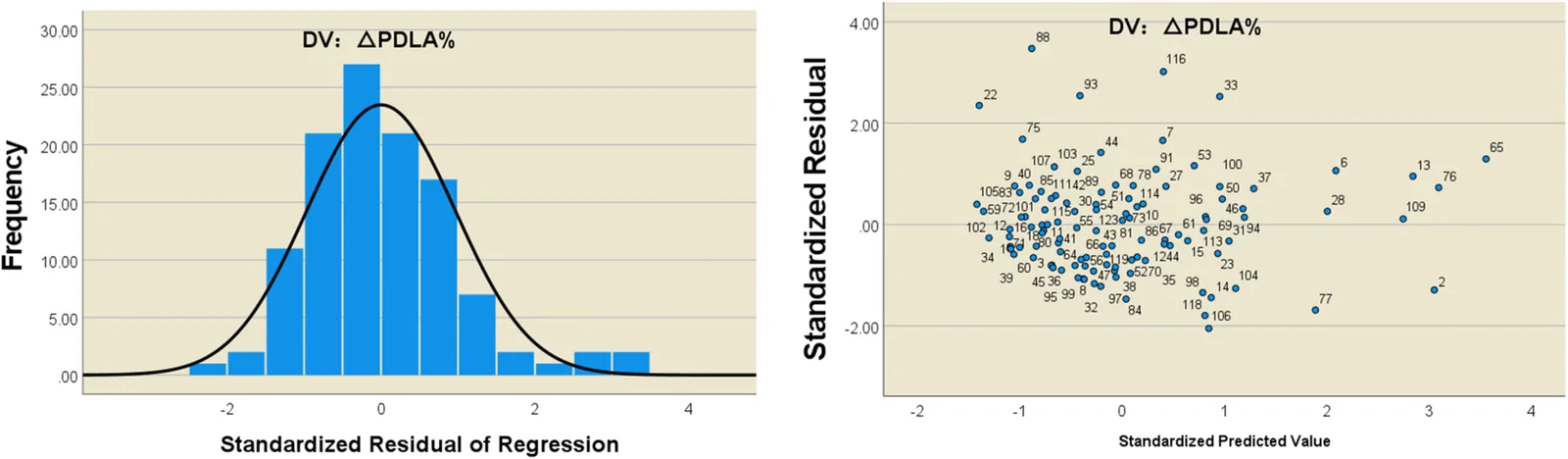
Histogram and scatter plot of standardized residuals for △PDLA% after removing outliers (Number of Cases = 114)

After excluding these two outliers, the robust regression model based on 114 observations remained highly significant (*F* = 11.397, *P* < 0.001), with the adjusted *R²* increased to 0.392. The independent positive association between ΔRL% and ΔPDLA% remained stable and prominent (B = 0.509, standardized *β* = 0.536, *t* = 6.595, *P* < 0.001). The pre-treatment U1-NA angle was also significantly positively associated with ΔPDLA% (B = 0.222, *P* < 0.05). Treatment duration (*P* = 0.082) and pre-treatment ∠ANB (*P* = 0.055) still approached statistical significance. Age, tooth extraction status and tooth position still showed no significant correlations with ΔPDLA% (all *P* > 0.05). Residual analyses verified the assumptions of normality and homoscedasticity. (Table 11; Fig. 7).

**Table 11:** Partial regression coefficients for ΔPDLA%

Independent Variable	B	β	*P*	DW	*R* ^2^	F
△RL%	0.509 (0.523)	0.536 (0.502)	<0.001** (<0.001**)	2.294 (2.160)	0.392 (0.342)	11.397 (9.521)
Pre-treatment U1-NA angle	0.222 (0.186)	0.257 (0.196)	0.004** (0.034*)			
∠ANB	0.448 (0.519)	0.176 (0.186)	0.055 (0.052)			
Treatment Duration	0.046 (0.050)	0.136 (0.134)	0.082 (0.096)			
Tooth extraction status	-0.154 (-0.345)	-0.012 (-0.024)	0.888 (0.781)			
Tooth position	0.381 (-0.372)	0.029 (-0.026)	0.693 (0.734)			
Age	0.052 (0.067)	0.069 (0.082)	0.396 (0.335)			

Multiple linear regression analysis; *: *P*<0.05; **: *P*<0.01

## Discussion

Deep learning-based CBCT-PDL segmentation system enables efficient and high-precision quantitative measurement of PDLA [6]. In the present study, this deep learning-assisted measurement framework was utilized to quantify post-treatment changes in PDLA (ΔPDLA) of bilateral maxillary central incisors. We systematically investigated the associations between patient-specific baseline clinical characteristics, orthodontic treatment-related variables, and ΔPDLA, while further assessing the independent correlation between orthodontically induced root resorption (ΔRL%) and ΔPDLA after adjusting for potential confounding factors.

Our statistical analyses entailed multiple subgroup comparisons and dichotomization of continuous variables based on clinically validated cut‑off thresholds, which may elevate the risk of type I error. Given the retrospective nature of the present study and its restriction to patients with complete paired pre‑ and post‑treatment CBCT datasets, potential selection bias cannot be entirely ruled out; accordingly, our findings should be interpreted with appropriate caution.

In the present subgroup analyses, several skeletal‑dental subgroups were excluded owing to extremely limited sample sizes, which may affect the generalizability of our findings. Specifically, Class III malocclusion (*n* = 6), skeletal Class III pattern (*n* = 6), patients with ∠ANB < 0° (*n* = 5), and low‑mandible‑angle cases (*n* = 3) were excluded from statistical comparisons to avoid unreliable between‑group differences caused by insufficient statistical power. Accordingly, our subgroup comparisons mainly focused on Class I and Class II malocclusion populations, as well as patients with average‑mandible‑angle and high‑mandible‑angle. However, in the multiple linear regression analysis, ∠ANB was included as a continuous variable, thus patients with ∠ANB < 0° were not excluded. Future research will expand the sample size to further validate the generalizability of the present findings.

### Patient-related factors

#### Demographic information (gender, age)

No statistically significant differences in pre-treatment PDLA were observed in different gender or age groups, indicating comparable initial levels of periodontal supporting tissue. Previous studies have reported that male patients experience greater root resorption following orthodontic treatment [13, 14], which may be attributed to gender-related hormonal differences. Estrogen inhibits osteoclast activity and promotes osteogenesis; therefore, females—particularly after adolescence—may have a protective effect against orthodontic force-induced bone resorption [15]. However, such a trend was not observed in this study.

Adolescent are in a period of rapid growth, characterized by active cellular metabolism and abundant vascularity. Mitophagy of periodontal ligament stem cells induced by orthodontic forces promotes alveolar bone remodeling [16]. Previous studies have also shown that at the same level of orthodontic force, the increase in cytokines and osteoclast markers is greater in adults than in adolescents, while tooth movement speed is slower in adults [17]. Therefore, under the same orthodontic stimulation, adolescent alveolar bone can undergo more effective adaptive remodeling, which may explain why adult patients exhibited greater ΔRL, ΔRL%, ΔPDLA, and ΔPDLA% compared with adolescents, with significant differences in ΔRL and ΔRL% between the two groups [8]. From a treatment safety perspective, orthodontic treatment is recommended to be performed during adolescence.

#### Pre-treatment U1-NA angle of maxillary central incisors

Patients were divided into protruded and non-protruded groups based on pre-treatment U1-NA angle, consistent with the classification criteria described earlier. The protruded group had higher ΔRL, ΔRL%, ΔPDLA, and ΔPDLA% than the non-protruded group, with a marginally significant difference in ΔPDLA. Mechanistically, when palatally retracting protruded maxillary central incisors, the root apex is prone to contact with the labial cortical bone plate, resulting in localized stress concentration. Excessive, particularly persistent, stress concentration within the PDL is a key mechanism underlying hyalinization and subsequent external root resorption [18], which may explain the observed differences in PDLA reduction.

Although univariate analysis did not identify significant differences in ΔPDLA% between patients with and without labial inclination of maxillary central incisors, multiple linear regression analysis revealed that pre‑treatment U1‑NA angle was significantly positively associated with ΔPDLA% (B = 0.186, standardized β = 0.196, t = 2.143, *P* < 0.05) after adjusting for potential confounding factors including age, ∠ANB, treatment duration and tooth extraction status. The relatively moderate standardized β value suggested that the independent effect of initial maxillary incisor inclination on periodontal‑alveolar bone remodeling was mild but statistically detectable. The discrepancy between univariate and multivariate outcomes may partly result from information loss caused by dichotomized grouping of incisor inclination in univariate analysis, as well as the masking effect of confounding variables. These findings preliminarily indicate that greater pre‑treatment U1‑NA angle may tend to correspond to increased ΔPDLA% following orthodontic treatment, implying that quantitative assessment of initial U1‑NA angle might serve as a potential baseline indicator for predicting the extent of post‑orthodontic periodontal‑alveolar bone remodeling, which could provide a reference for individualized orthodontic force control and clinical risk evaluation.

#### Skeletal pattern

Sagittally, patients with Angle’s Class II malocclusion or skeletal Class II exhibited higher ΔRL, ΔRL%, ΔPDLA, and ΔPDLA% compared to those with Angle’s Class I malocclusion or skeletal Class I, respectively. These findings are consistent with those of previous studies [19, 20]. Notably, Angle’s classification and ∠ANB may exhibit collinearity as both reflect sagittal occluso‑skeletal relationships. However, they represent distinct clinical phenotypes: Angle’s classification indicates dental‑occlusal features, whereas ∠ANB quantifies skeletal discrepancy. In this study, separate subgroup analyses rather than combined regression modeling were performed to avoid multicollinearity. This strategy helps differentiate dental compensation from true skeletal effects, although their interdependence should be acknowledged when interpreting the findings. Further subgroup analysis stratified by ∠ANB demonstrated that patients with 0<∠ANB ≤ 3° exhibited lower ΔPDLA and ΔPDLA% than those with ∠ANB > 3° (*P* < 0.05).

In terms of vertical skeletal patterns, average‑mandible‑angle patients showed higher ΔRL and ΔRL% but lower ΔPDLA and ΔPDLA% compared with high‑mandible‑angle patients. These findings imply that ΔRL and ΔPDLA may represent distinct clinical manifestations, and alveolar bone remodeling potential varies substantially across different skeletal morphotypes. In patients with 0<∠ANB ≤ 3° or average‑mandible‑angle patterns, relatively harmonious jaw relationships may facilitate favorable alveolar bone remodeling, which could partially counteract the adverse impacts of root resorption. This compensatory effect may maintain periodontal ligament stability and prevent excessive reduction in PDLA. By contrast, high-mandible-angle patients commonly feature posterior mandibular rotation, increased lower-facial height, thinner alveolar bone and potentially decreased bone density, which may compromise alveolar bone remodeling capacity. For skeletal Class II patients with maxillary prognathism, anterior tooth retraction necessitates extensive root displacement that may exceed the adaptive capacity of alveolar bone. In both scenarios, alveolar bone remodeling may fail to keep pace with orthodontic tooth movement, increasing risks of labial bone dehiscence or fenestration. Accordingly, relatively greater ΔPDLA and ΔPDLA% may be observed even with moderate root resorption. This finding offers tentative implications for individualized orthodontic treatment planning.

From a clinical perspective, for patients with skeletal Class I or average‑mandible‑angle patterns, clinical attention should focus on minimizing root resorption, such as by optimizing orthodontic force magnitudes and directions to avoid excessive or reciprocating tooth movement. For patients with skeletal Class II or high‑mandible‑angle patterns, priority should be given to strategies preserving and enhancing alveolar bone remodeling capacity. In particular, patients with thin labial cortical bone plates may benefit from adjunctive bone augmentation procedures to improve local bone quality and remodeling potential.

In the multivariate regression model, pre‑treatment ∠ANB exhibited a marginally significant positive association with ΔPDLA% (B = 0.448, standardized β = 0.176, t = 1.943, *P* = 0.055). This borderline significance, together with the relatively low standardized β value, suggested a weak potential independent effect of skeletal sagittal discrepancy on post‑orthodontic periodontal‑alveolar bone remodeling, which requires further validation with larger sample sizes. Notably, sagittal skeletal discrepancy was assessed solely via the ANB angle in the present study, which may not fully capture comprehensive skeletal characteristics. Therefore, incorporating multiple skeletal indicators such as Wits analysis in future research will help improve the precision of skeletal evaluation.

#### Chronic periodontitis

Current research on orthodontic treatment in patients with chronic periodontitis suggests that orthodontic treatment with regular periodontal maintenance does not result in increased alveolar bone loss and may even enhance alveolar bone attachment levels after anterior tooth intrusion and retraction [21–24]. Animal studies have shown that when periodontal inflammation is well-controlled, the application of orthodontic forces helps increase alveolar bone volume and improve bone quality [25]. Combined orthodontic-periodontal therapy can reposition protruded anterior teeth with subgingival pockets, reduce periodontal probing depth, and increase clinical attachment levels [24]. Thus, orthodontic treatment can achieve satisfactory aesthetic, occlusal, and functional objects, and combined orthodontic-periodontal therapy is an ideal treatment option for patients with chronic periodontitis [22].

Although the sample size of patients with chronic periodontitis in this study was small (*n* = 7), the overall trend showed that the chronic periodontitis group had smaller ΔPDLA and less root resorption, which may be attributed to the application of gentler orthodontic forces and stricter periodontal maintenance in these patients during treatment. Therefore, chronic periodontitis is not a risk factor for PDLA reduction in patients with well-controlled periodontal inflammation. Future studies should expand the sample size of patients with chronic periodontitis to eliminate the interference of insufficient statistical power and further validate this finding.

### Treatment-related factors

#### Extraction therapy

Most previous studies indicate that tooth extraction increases the risk of root resorption [13, 26–28], while others have found no association between extraction therapy and root resorption [29]. In the present study, univariate analysis revealed that extraction therapy was associated with greater root resorption and PDLA reduction, whereas multiple linear regression further demonstrated that tooth extraction was not an independent influencing factor for ΔPDLA% after adjusting for confounding variables including age, pre‑treatment ∠ANB, treatment duration and initial incisor inclination. Such inconsistency between univariate and multivariate findings may be explained by confounding effects: patients receiving extraction treatment generally present with more severe skeletal‑dental discrepancies, longer treatment duration, greater magnitude of anterior tooth retraction, which collectively contribute to PDLA loss rather than extraction per se. Therefore, extraction‑related periodontal changes may be secondary to complex skeletal‑dental characteristics and treatment‑related factors rather than a direct consequence of tooth removal.

Clinically, for orthodontic patients with borderline extraction indications, comprehensive assessment of their skeletal‑dental patterns, treatment duration, and alveolar bone condition is required, and extraction should be performed cautiously not because extraction itself directly impairs PDLA, but to avoid the potential cumulative effects of associated confounding factors (e.g., more extensive tooth movement, prolonged treatment) on periodontal health. For patients undergoing extraction, preoperative CBCT evaluation of root length and anterior alveolar bone thickness and height is recommended. During orthodontic intervention, segmented tooth movement and light‑force sliding mechanics should be adopted to minimize periodontal injury. Additionally, temporary interruption of active treatment for 2 to 3 months may help mitigate progressive root resorption once radiographic signs are detected [30].

#### Orthodontic treatment duration

The association between orthodontic treatment duration and root resorption remains controversial in existing literature. Segal GR et al. concluded through a meta-analysis that longer treatment durations are associated with an increased risk of root resorption [31]. In contrast, a prospective study by Henrik et al. failed to identify any correlation between apical resorption and treatment duration [13], and clinical guidelines also indicate that the duration of active orthodontic treatment is not an independent risk factor for the severity of root resorption [14].

In the present study, patients with orthodontic treatment duration exceeding 2 years exhibited a trend of increased root resorption compared with those with shorter treatment durations, though this difference did not reach statistical significance. Regarding periodontal ligament area changes, the difference in absolute ΔPDLA approached statistical significance, while the difference in relative ΔPDLA% was statistically significant. Consistent with these subgroup findings, multivariate linear regression analysis further showed that orthodontic treatment duration was marginally positively associated with ΔPDLA% (β = 0.136, *P* = 0.082). This borderline P-value, combined with the relatively low standardized β value, suggests that longer treatment duration may have a weak potential positive effect on PDLA reduction. The observed trend may be attributed to the cumulative mechanical stress on periodontal tissues during prolonged orthodontic tooth movement, which could gradually compromise alveolar bone remodeling capacity and lead to progressive PDLA reduction, even if the associated root resorption does not reach statistical significance. Therefore, optimizing orthodontic treatment efficiency should be prioritized as a clinical strategy to reduce PDLA loss: this includes avoiding overtreatment, minimizing frequent modifications to treatment plans, reducing unnecessary tooth movement and anchorage loss, and shortening the overall treatment duration as much as possible. These measures may help mitigate the cumulative adverse effects of prolonged mechanical stimulation on periodontal tissues, particularly for patients who are expected to have a treatment duration exceeding 2 years, thereby maintaining periodontal ligament stability during orthodontic treatment.

This study only evaluated the changes in PDLA before and after treatment, and focused on the risk factors contributing to the reduction in the PDLA of maxillary central incisors during active orthodontic treatment. Periodontal tissue remodeling and repair is a continuous physiological process. After the removal of orthodontic force, the PDLA may improve along with the reconstruction of occlusal function and the restoration of periodontal tissue homeostasis. Therefore, the decreased PDLA after treatment should not be regarded as a permanent loss of periodontal tissue.

Univariate subgroup analyses in the present study demonstrated that patients with extraction therapy and treatment duration longer than 2 years exhibited significantly greater ΔPDLA and ΔPDLA%. Notably, however, multiple linear regression analysis adjusting for interrelated baseline confounders revealed that none of these commonly presumed risk factors exerted an independent predictive effect on ΔPDLA. This apparent discrepancy between univariate and multivariate outcomes can be explained by cumulative confounding effect. The increased periodontal damage observed in these subgroups arises from the combined influence of multiple interconnected clinical characteristics rather than from any single isolated variable alone. Consequently, periodontal ligament loss following orthodontic treatment represents a multifactorial outcome shaped by complex interactions among patient-specific skeletal-dental features. From a clinical perspective, those conventional indicators can still serve as rapid initial screening tools to stratify patients requiring intensified periodontal monitoring during orthodontic treatment.

## Conclusion

This study compared changes in PDLA and RL of maxillary central incisors before and after orthodontic treatment, evaluated patient-related and treatment-related risk factors. The results demonstrated that the reduction in PDLA of maxillary central incisors is associated with multifactorial interactions involving skeletal (∠ANB, FMA), U1-NA angle, and treatment-related factors (extraction therapy, treatment duration). Factors associated with marked changes in RL and PDLA are not entirely identical. Multiple linear regression analysis confirmed a strong correlation between ΔRL% and ΔPDLA%. This indicates that RL and PDLA serve as independent yet closely related indicators for assessing root and periodontal health during orthodontic treatment.

The deep learning model developed for automated PDL segmentation based on CBCT images represents a novel and reliable method to quantify PDLA of maxillary central incisors in orthodontic research. This technique is conducive to assessing the safety of tooth movement and guiding individualized treatment planning. In this regard, comprehensive pretreatment evaluation of individual skeletal and dental characteristics, instead of depending on single indicators, is fundamental to personalized risk assessment and clinical decision-making in orthodontics.

## Data Availability

The datasets used and/or analysed during the current study available from the corresponding author on reasonable request.

## Abbreviations

PDL: Periodontal ligament
PDLA: Periodontal ligament area
CBCT: Cone beam computed tomography
RL: Root length
ICC: Intraclass correlation coefficient
